# Recruiting the Immune System against Pathogenic Bacteria Using High-Affinity Chimeric Tags

**DOI:** 10.1021/acs.bioconjchem.4c00291

**Published:** 2024-10-14

**Authors:** Yael Belo, Einav Malach, Zvi Hayouka

**Affiliations:** Institute of Biochemistry, Food Science and Nutrition, The Robert H. Smith Faculty of Agricultural, Food & Environment, The Hebrew University of Jerusalem, Rehovot 76100, Israel

## Abstract

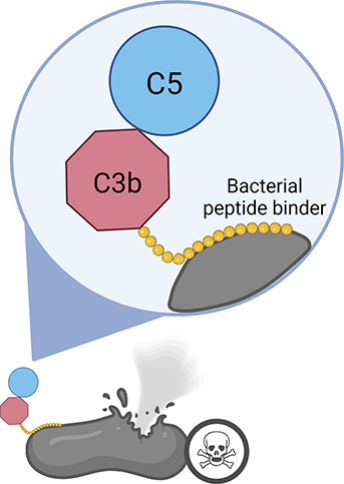

The immune system plays a critical role in protecting the host against pathogens. However, mechanisms for evading the immune system have evolved in pathogens, altering their surface proteins or causing the expression of enzymes that interfere with the immune response. These strategies cause pathogens to escape detection and destruction by the immune system, thereby inducing severe infections. Thus, there is a critical need to develop new chemical tools to recruit the immune system against evading pathogens. Here, we describe a novel strategy for targeting pathogens, by labeling them with a chimeric agent that comprises a peptide bacterial binder, conjugated to an immune-protein tag that is recognizable by the complement system, thereby recruiting the immune system against the targeted pathogen. The chimeric tag was developed by conjugating the peptide bacterial binder with the C3b complement system activating protein. We showed that the chimeric C3b tag preserved its activity and was able to bind the C5 complement protein with strong binding affinity. Using this approach, we have demonstrated that the chimeric agent was able to eradicate 90% of complement-resistant *E. coli* bacterial cells. By showing enhancement of complement sensitivity in complement-resistant pathogens, this work demonstrates the basis for a new therapeutic approach for targeting pathogenic bacteria, which could open a new era in the development of selective and effective antimicrobial agents.

## Introduction

**Figure 1:**
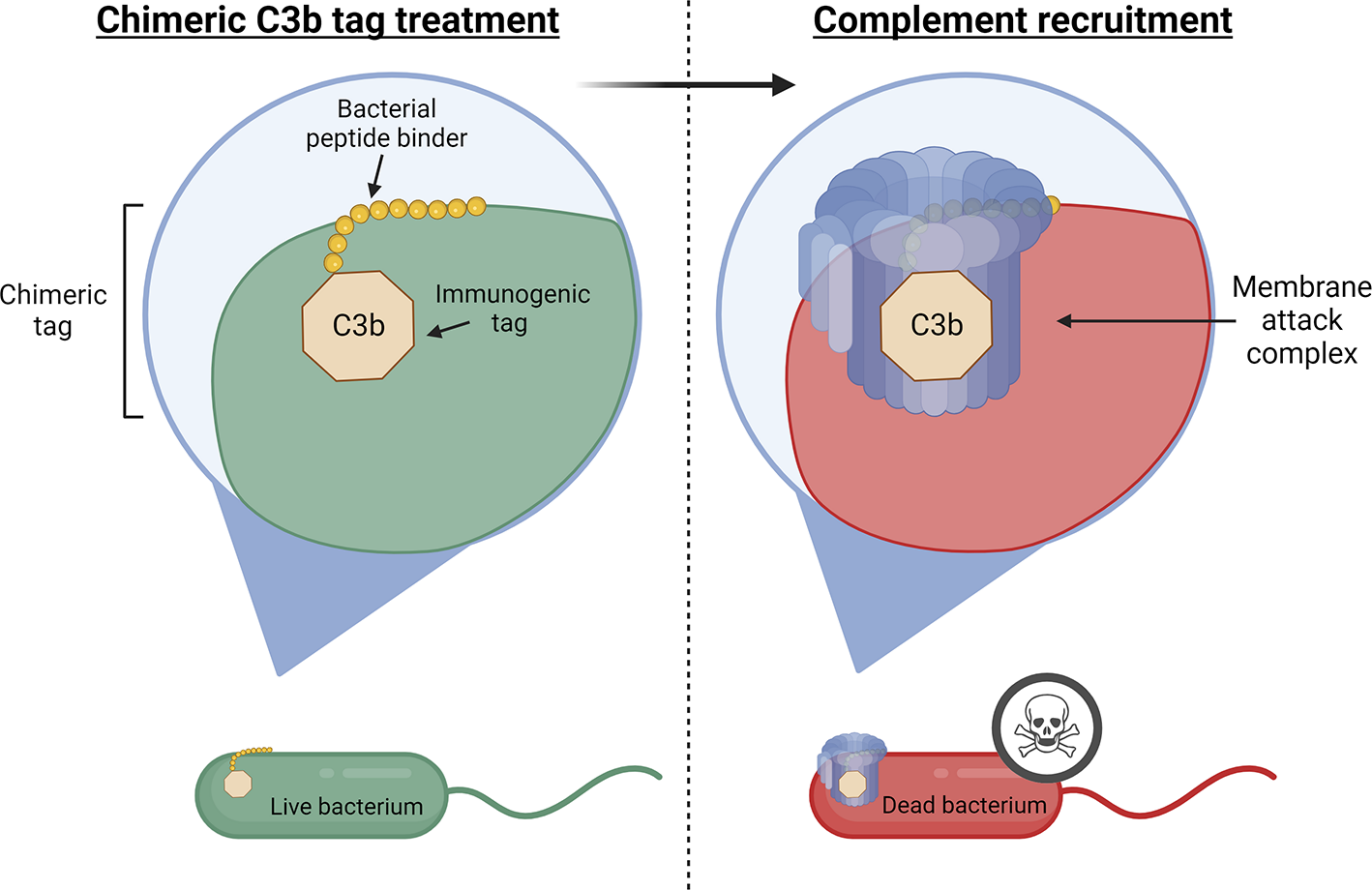
**Representative illustration of the designed approach**. Utilizing a chimeric C3b tag, we targeted and treated complement-resistant pathogenic bacteria, initiating complement system recruitment. Subsequently, this process induced the formation of the membrane attack complex, leading to efficient bacterial clearance.

The protection of most organisms against invading pathogens relies heavily on the crucial and variable functions of their immune systems.^1,2^ The human immune system has an elaborate network of cascades to fight microbial intruders.^3,4^ Within this network, the complement system acts as a mediator in the elimination of pathogens, by constituting one of the earliest and most important responses to pathogen infections.^5,6^ Complement activation occurs through one of its three activation pathways, namely, the classical, alternative and lectin pathways.^7^ Within the alternative complement cascade, C3 convertase activates the protein C3 through cleavage of a single peptide bond in C3, converting it into C3a and C3b protein fragments.^8,9^ C3b carries a thioester reactive group, that attaches the fragment to bacterial cell membranes by covalently binding hydroxyl groups found on the membrane.^10^ Due to rapid hydrolysis of the reactive thioester group, most active C3b molecules fail to attach to the foreign matter.^11^ A high density of C3b molecules (*e.g*., on complement-opsonized foreign-matter surfaces) forms high-affinity binding sites for the C5 protein.^10^ C5 is further cleaved by C5 convertases into C5a and C5b fragments,^12^ whereupon C5b activates the alternative complement cascade by collaborating with the complement proteins C6 through C9, to create the Membrane Attack Complex (MAC).^13^ MAC induces pores in the bacterial cell membrane by injecting itself into the membrane, resulting in lysis.^14^ Complement-dependent bacterial eradication is one of the most immediate ways of killing an invading pathogen bacterium, and it is much faster than phagocytosis and the subsequent intracellular eradication.^4^ The fact that mechanisms to resist steps of the complement cascade have evolved in pathogenic bacteria points to its significant role in human defense against pathogens.^15,16,3^ Such resistance to complement elements is also associated with the composition of the bacterial lipo-polysaccharide (LPS). LPS is a vital component of the outer membrane in Gram-negative bacteria, contributing to its structural integrity.^17^ It consists of three distinct domains: the membrane-embedded lipid A, core oligosaccharides, and the O-antigen polysaccharides. Bacterial strains that have all three regions were defined as smooth LPS, while those lacking the O-antigen were termed rough LPS.^18^ Virulence traits, *i.e*., resistance to complement and phagocytosis or to killing by macrophages and neutrophils, might be affected by the length and structure of the O-poly-saccharides. These findings demonstrate the urgent need to develop chemical tools to recruit the immune system toward immune resistant pathogens and abort the pathogenic traits that allow evasion.^19^ Several examples for enhancing complement activation toward pathogens were reported in the literature. A recently developed biological synthetic approach termed antibody recruiting molecules (ARMs) mediates immune clearance through small molecules capable of enhancing antibody attachment to bacteria, as well as to disease-relevant cells or viruses.^19−21^ Rice and colleagues proposed a strategy to enhance complement activation against *Neisseria*,^22^ a bacterium that inhibits complement activation by binding to factor H (FH). The researchers created FH-Fc, a fusion protein combining FH’s bacterial binding domains with the Fc region of IgG. FH-Fc showed enhanced complement-dependent killing *in vitro* and in animal models for both gonorrhea and meningococcal bacteremia. In a conceptually related study, a click chemistry reaction was used to attach complement C3b to *E. coli* bacteria.^23^ These C3b-*E. coli* conjugates were exposed to human neutrophils, triggered phagocytosis, and led to the destruction of bacterial cells through complement activation. In the current study, instead of focusing on a specific protein found on the surface of a particular pathogen or a particular structural component of the bacterial cell surface, our approach targets bacterial membranes using a broader strategy. We have developed a random cationic peptide binder comprising Lysine and Isoleucine amino acids, which interact effectively with the bacterial membrane without affecting bacterial proliferation. Unlike small and specific molecules, such as ARMs, our peptide binder is a mixture of 1028 different peptides, with each variant containing the same two amino acids but in a different order along the peptide chain. Our strategy does not rely on the recruitment of antibodies for immune activation. Instead, we have developed a chimeric tag, composed of a bacterial binder motif, conjugated to immunoprotein that recruits the immune system toward the targeted bacteria (Figure 1). This allowed us to tag bacteria with our chimeric agent, to induce enhanced complement activation toward labeled bacteria. We show that our novel chemical tools can label pathogens and prevent them from evading detection and destruction by the immune system *in vitro*. We propose that this new general antievasion strategy can be used effectively to eradicate an unlimited variety of pathogens in a variety of hosts.

## Results

### Developing the Chimeric Tag

Our group has previously developed a new type of antimicrobial peptide, termed random peptide mixture (RPM), comprising hydrophobic and cationic amino acids. We have demonstrated that RPMs exhibit strong antimicrobial activity toward both Gram-negative and Gram-positive bacteria, including against antibiotic-resistant bacteria.^24−27^ As we have shown, the chain length of an RPM significantly affects its antimicrobial activity. We reported strong and broad antimicrobial activity of 20-mer RPMs, compared to 10-mer RPMs, which displayed very low (if any) antimicrobial activity, although high affinity to the bacterial membrane was observed in both RPM types.^25^ The 10-mer RPM comprised a 1:1 molar combination of Isoleucine and Lysine (IK). Here, we sought to identify an RPM that can function as high-affinity binder but nonetheless has little antimicrobial effect on its own. We hypothesized that the high positive charge of a 10-mer IK RPM increases the binding affinities to the bacterial membranes. We therefore first synthesized 10-mer IK labeled with 5(6)-carboxyfluorescein and measured affinities to several pathogens. We observed very high binding affinities to *E. coli R* and *S* bacteria (Figure S1, Supporting Information). In addition, we examined the effect of 10-mer IK on the growth of *E.coli* (Figure S2, Supporting Information) and observed minimal growth-inhibition. Therefore, a 10-mer IK RPM was further explored as a model of a peptide bacterial binder.

### Selecting the Immune System Activation Tag

**Figure 2:**
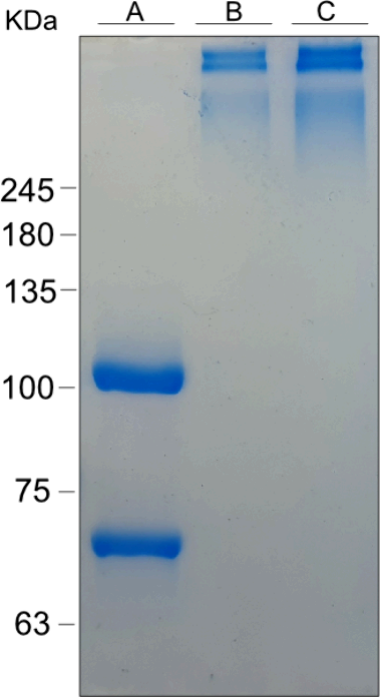
**Fluorescent peptide bacterial binder conjugate to C3b protein**. 10% SDS page of the conjugated fluorescent peptide bacterial binder to C3b. C3b molar mass is 176 kDa. C3b is composed of two chains, the α chain is 101 kDa and the β chain is 75 kDa. (A) C3b before conjugation to the peptide bacterial binder runs as its two subunits. (B) C3b conjugated to Fluorescein-peptide bacterial binder. (C) C3b protein conjugated to itself.

Since an important component of the innate immune system is the complement-dependent assembly of a protein complex on the bacterial membrane, we aimed to mimic this system. To achieve this, we conjugated C3b, one of the main proteins of the complex, to the peptide bacterial binder, using glutaraldehyde.^28,29^ As can be observed in Figure 2, following the reaction, the two subunits of C3b were coupled together, and a variable number of the binder molecules were conjugated to each C3b molecule. Since the binder simulates a membranal anchoring site, this implied that each C3b unit contains multiple binding sites for the target bacteria.

**Figure 3:**
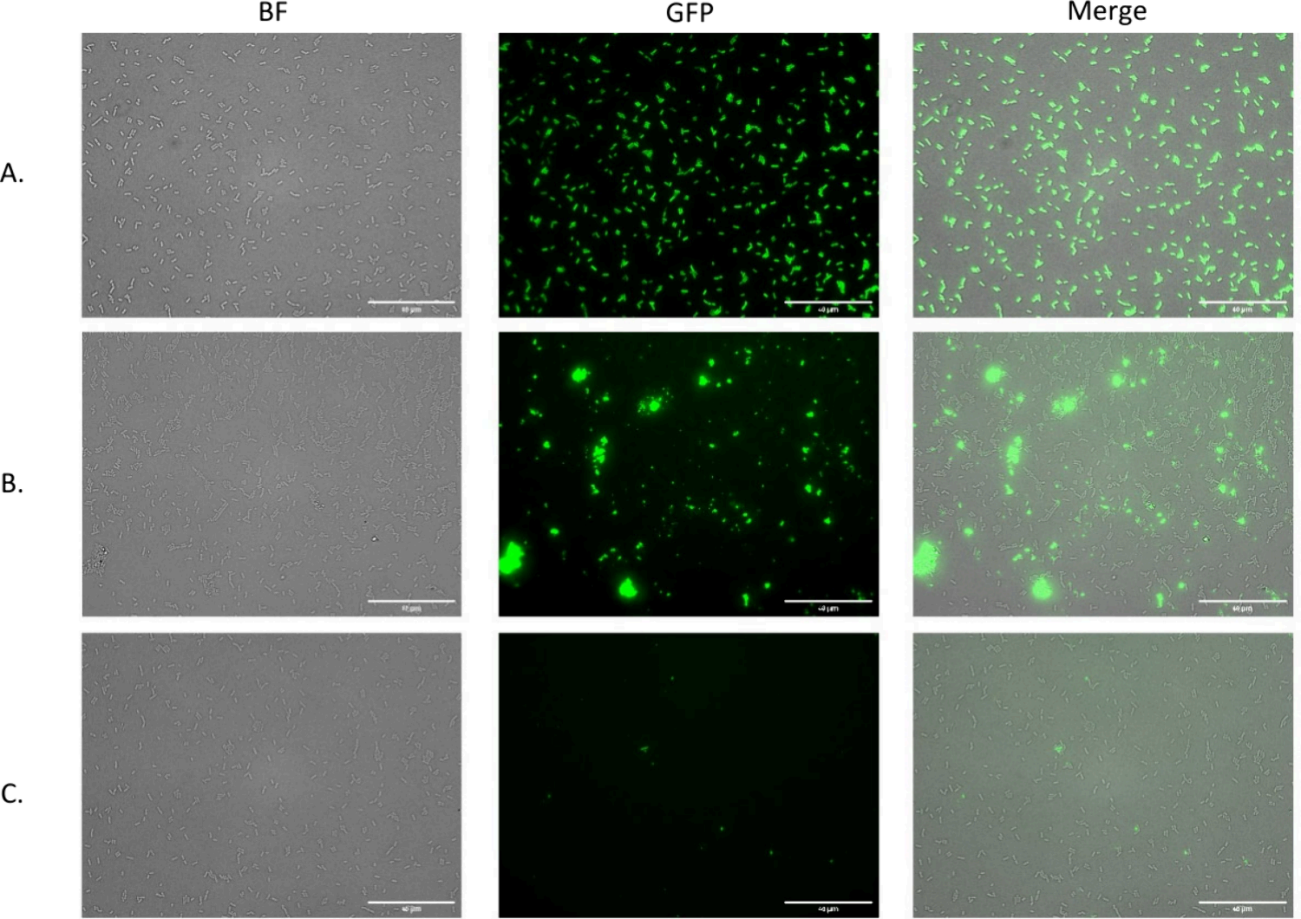
**Fluorescent chimeric C3b tag binds*****E. coli S***. EVOS microscope images of bound chimeric C3b tag to *P4-NR smooth E. coli* bacteria. Bacteria were grown to O.D. = 1 and incubated together with the fluorescent chimeric tag for 30 min at 37 °C in PBS. (A) Control sample which contained *P4-NR smooth E. coli* bacteria dyed with Syto9. (B) Binding to 0.22 mg/mL fluorescent chimeric tag. (C) Binding to 0.022 mg/mL fluorescent chimeric tag. Images shown were taken at a magnification of 40×.

We performed LC-MS/MS analysis in order to characterize the chimeric tag structure. We identified six C3b positions conjugated to the 10-mer IK peptide bacterial binder, demonstrating that each complex contains multiple potential binding sites for the target bacteria (Figure S3, Supporting Information). The amino acids that were modified are aspartic acid, glutamic acid, and glutamine. The final concentration of the chimeric tag, determined using the Bradford reagent, was 0.22 mg/mL. Since the binders were shown to have high affinity to *E. coli R* and *S* bacteria, the multitude of such binders on one C3b unit was projected to grant excellent binding affinity to those target bacterial pathogens. To examine the binding to *E. coli* bacterial cells of the new chimeric C3b tag, we synthesized fluorescently labeled 10-mer IK peptide binders and conjugated them to C3b. As demonstrated in Figure 3, the chimeric tag binds *E. coli* bacterial cells in a strong concentration-dependent manner. In addition, we examined the fluorescent tag alone, without bacteria, to rule out probe aggregation (Figure S4, Supporting Information). The fluorescent chimeric tag units remained well-dispersed, indicating that the observed clumping is due to bacterial aggregation.

### A Chimeric C3b Tag Binds Complement C5 Protein

**Figure 4:**
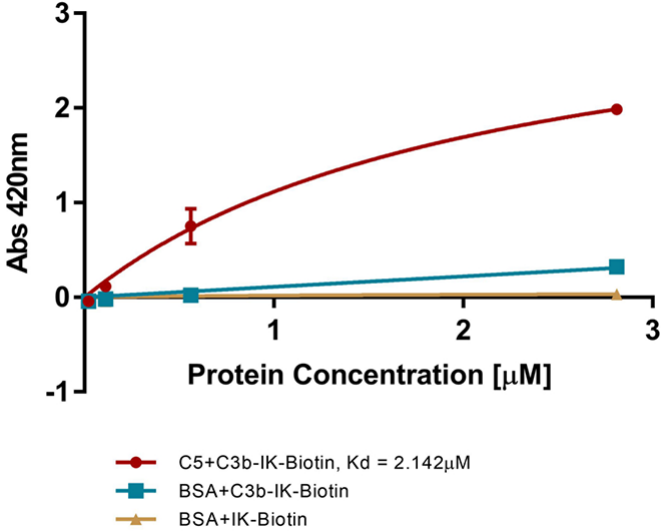
**10-mer IK-C3b complex binds C5**. C5 was fixed onto an ELISA plate, and Chimeric C3b-Biotin was added in a serial dilution (red). Mean ± SEM (*n* = 2). Negative controls: blue: C5 was replaced with BSA; yellow: binding of IK-Biotin to BSA is shown (curve, overlaps with *x*-axis). Binding was examined using anti streptavidin-HRP antibody at 420 nm. This is a representative result out of three biological repeats.

It was recently shown that at very high C3b density, MAC formation can be detected even in the absence of C5 activating enzymes.^30^ To examine the ability of the chimeric C3b tag to activate the complement alternative pathway, we conducted an enzyme-linked immunosorbent assay (ELISA). To that end, we coupled a biotin molecule to the peptide bacterial binder and conjugated it to C3b by using glutaraldehyde. ELISA microplates coated with the C5 protein and incubated with the binder-biotin-C3b conjugate in serial dilutions. As shown in Figure 4, the chimeric tag showed a specific and strong binding to C5 (measured using streptavidin-HRP antibody), with an average K_*D*_ of 1.63 ± 0.53 μM (IK-IgG and BSA controls showed no binding), indicating the potential of the conjugate to induce complement system activation.

### A Chimeric C3b Tag Induces Complement Sensitivity in *E. coli* Bacteria

**Figure 5:**
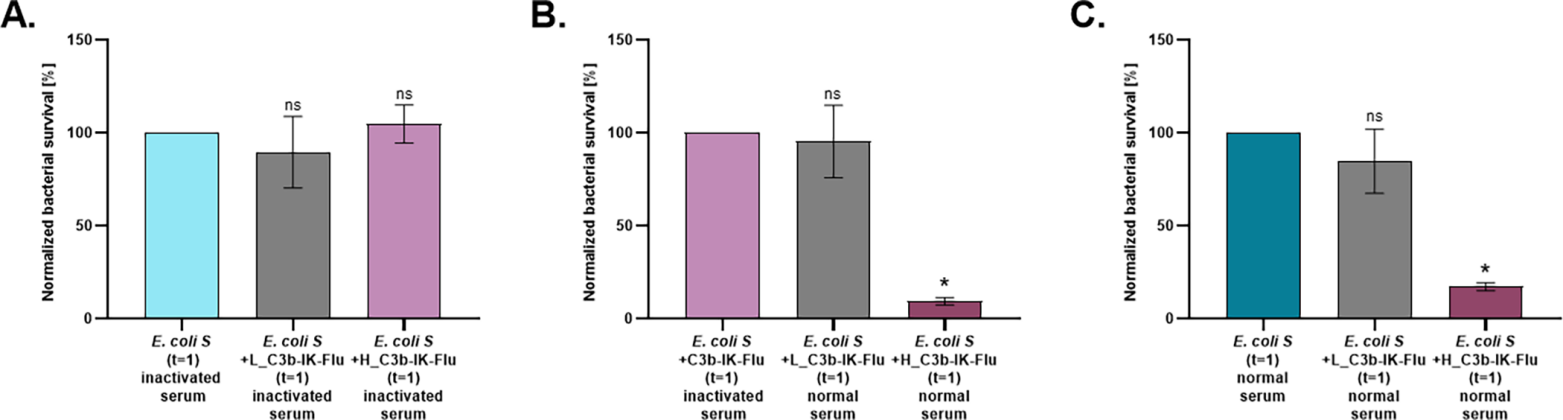
**C3b chimeric tag induces complement sensitivity of*****E. coli S*****bacteria**. Bacteria were incubated with normal human serum or inactivated serum for 1 h at 37 °C. Samples were microdiluted and plated on LB agar plates. (A) *E. coli S* and chimeric C3b tag treated *E. coli S*, in inactivated serum. (B) Treated *E. coli S* in inactivated or normal serum. (C) Nontreated *E. coli S* and chimeric C3b tag treated *E. coli S*, in normal serum. The chimeric tag treatment is presented in two concentrations: H_C3b-IK-Flu represents the high concentration (0.22 mg/mL), while L_C3b-IK-Flu indicates a 10-fold lower concentration (0.022 mg/mL). Mean ± SEM (*n* = 3). *P < 0.05.

To evaluate the ability of the chimeric C3b tag to activate the complement system against bacteria, we examined whether the tag can induce eradication even of a bacterium that is serum-resistant. First, a serum-sensitivity assay was designed, employing *E. coli* strains with varying vulnerability to normal serum. Such sera contain the proteins of the complement system that form the MAC complex, causing bacterial killing, and hence can be used for probing complement-sensitivity. The rough *E. coli* strain (hereafter *E. coli R*), which is known to be vulnerable to the complement system, and the smooth *E. coli* strain (hereafter *E. coli S*), known to have complement-resistance, were incubated either with normal human serum or with heat-inactivated human serum (in which the heat is known to eliminate the complement proteins). As expected, the results showed complete eradication of *E. coli R*, whereas *E. coli S* showed great resistance toward the serum (Figure S5, Supporting Information), while no eradication activity was observed in the presence of inactivated serum (Figure S6, Supporting Information). For the serum sensitivity assay, *E. coli* bacterial cells were incubated with a 0.22 mg/mL or a 10-fold lower concentration (0.022 mg/mL) of the chimeric C3b tag for 30 min. All bacteria samples were then incubated with 10% normal human serum or with heat inactivated serum as a negative control. After 1 h of incubation, microdilution plating was carried out on an LB-agar plate. Finally, colonies counting was performed to evaluate the eradication effectiveness. Based on our results showing no antimicrobial activity of the 10-mer IK binder, and the fact that C3b is not toxic to bacteria when appearing in isolation of the complement system, we expected the chimeric tag to have no inherent antimicrobial activity. This was confirmed when we examined the tag’s inherent activity, *i.e*., regardless of any additional complement effects, in deactivated serum (where complement activity had been neutralized), on the complement-resistant strain *E. coli S*; we used the resistant strain so that should killing be observed, it could not be attributed to complement activity, to which, as we have shown, the strain is resilient. As expected, the bacterial loads (CFU/mL) of chimeric C3b tag-treated and nontreated bacteria were similar in inactivated serum, at 100% and 104.72% (Figure 5A), demonstrating that the tag has no antimicrobial effects of its own. We also aimed to demonstrate that the chimeric C3b tag killing capability relies on its interaction with active complement proteins, leading to the formation of MAC. Therefore, we incubated chimeric C3b tag-treated *E. coli S* in normal serum and inactivated serum separately. We observed that only 6% of the C3b tagged cells survived the normal serum treatment, compared to 100% survival of the inactivated serum-treated cells (Figure 5B). Finally, we compared the bacterial loads of chimeric C3b tag-treated *E. coli S* to nontreated *E. coli S*, that were both incubated in normal serum. The bacterial load of the treated *E. coli S* was reduced by 90% compared to the nontreated bacteria (Figure 5C), demonstrating the superb killing activity of the tag and, most importantly, its ability to circumvent the evasion mechanism of a strain resistant to complement-mediated killing. Conclusively, the treatment of a lower concentration exhibited significantly lower activity compared to the higher concentration, indicating a concentration-dependent activity of the chimeric tag.

## Discussion

Our overarching goal was to test a versatile approach capable of tagging diverse cell types, including bacteria and fungi, in order to recruit the immune system to fight them. This ambitious goal was aimed at harnessing the power of the immune system against a specific microbial target and combating a wide range of cell-based threats, irrespective of their nature or origin. In the current study we established a new approach to redirect the immune complement response toward complement-resistant bacterial pathogens. The bacterial peptide binder we have selected was shown to possess strong binding affinity toward a diverse array of bacterial species, including both Gram-negative and Gram-positive bacteria. Here, we have shown that conjugating a bacterial peptide binder to the complement protein C3b (chimeric tag) results in a number of binder molecules attaching to the tag. We postulate that each such complex comprises multiple potential binding sites for the target bacteria. These multivalent complexes may thus have the potential to enhance the binding affinity of the tag (C3b in this case) for the targeted bacterial pathogen. We have further demonstrated that the fluorescent chimeric C3b tag (Fl′-chimeric tag) binds *E. coli S* in a concentration-dependent manner and have provided evidence that this chimeric tag exhibits good binding affinity to another important complement system protein, C5. Based on these observations, it is likely that our complex has activated the alternative pathway cascade, leading to the formation of the Membrane Attack Complex (MAC). We have demonstrated that incubation of the chimeric tag with *E. coli S* significantly heightened its susceptibility to serum treatment, compared to nontreated *E. coli S* or the bacteria when treated in inactivated serum. This implies that the presence of the chimeric tag enhances the sensitivity of the resistant bacteria to the bactericidal effects of the serum, ostensibly through increased complement-mediated killing. The new methodology presented holds the potential to pave the way for novel treatments targeting diverse bacterial infections, prompting further research to explore and further utilize its application. Such ongoing research will contribute to the development of innovative therapies for combating bacterial infections and addressing the challenges posed by the ability of bacteria to evade the immune system and to proliferate in the host.

## Materials and Methods

### Bacterial Strains

*E. coli* strains *P4-NRΔgalU rough* (*E. coli R*) and *P4-NR smooth* (*E. coli S*) were kindly provided by Nahum Shpigel. *E. coli* strain *P4-NRΔgalU rough* (*E. coli R*) contains kanamycin resistance. All bacterial cells used in this study were stored in 25% glycerol at 80 °C.

### Peptide Synthesis

Peptides were synthesized by a standard Fmoc-based solid-phase peptide synthesis (SPPS) on Rink Amide resin (substitution 0.6 mmol/g) using a peptide synthesizer (Liberty Blue). Amino acids were dissolved with dimethylformamide (DMF) to a final concentration of 0.2 M. The Fmoc deprotection step was conducted by 20% piperidine in DMF. For conjugation of 5-(6)-carboxyfluorescein to 10-mer IK Random Peptide Mixture (RPM), 5-(6)-carboxyfluorescein (4 equiv relative to the overall loading of the resin) was dissolved in 92% DMF and 8% dichloromethane (DCM). This solution was mixed with hydroxybenzotriazole (HOBt; 4 equiv), diisopropylethylamine (DIEA; 4 equiv), and *N*,*N*′-diisopropylcarbodiimide (DIC; 4 equiv) overnight with agitation. The 10-mer IK random peptide mixture (RPM) was conjugated to 5-(6)-carboxyfluorescein through its amine group. Upon completion of the conjugation, the peptides were cleaved from the resin by adding a solution containing 95% trifluoroacetic acid (TFA), 2.5% double-distilled water (DDW) and 2.5% triisopropylsilane (TIPS) and stirred for 3 h. The mixture was then filtered, and the peptides precipitated by the addition of 40 mL of cold diethyl ether to the TFA solution and centrifuged. The supernatant was then removed, and the peptide pellet dried, dissolved in 20% acetonitrile in DDW, frozen with liquid nitrogen, and lyophilized. The synthesis was validated by MALDI-TOF mass spectrometry.

### C3b Conjugation to 10-mer IK Using Glutaraldehyde and Complex Concentration Determination

Human C3b protein was purchased from complement technology and mixed together with 10-mer random IK peptide mixture conjugated to a fluorescein molecule in a molar ratio of 1:29.5. Glutaraldehyde was added over a 1–2 min period, dropwise and mixed gently with a magnetic stir for 1 h at room temperature. After 1 h 10 mM Glycine was added to stop the reaction. This was followed by overnight dialysis vs 0.5 L PBS buffer at 4 °C, freezing in liquid nitrogen, and maintaining at −80 °C. The final concentration of the chimeric tag, determined using the Bradford reagent (Bio-Rad), was 0.22 mg/mL. A calibration curve was created by using serial dilutions of bovine serum albumin (BSA). Each diluted BSA sample or C3b chimeric tag sample (50 μL) was mixed with 150 μL of 4× diluted Bradford reagent in a well of a 96-well plate. After a 10 min incubation, optical density (O.D.) readings were taken at 595 nm using a Tecan plate reader.

### LC-MS/MS Analysis

The conjugates were digested using trypsin, analyzed by LC-MS/MS on a Q-Exactive HF (Thermo), and identified using Discoverer software against the human C3b sequence from the Uniprot database, the human serum database from Uniprot, and a decoy database.

### Fluorescent C3b-Chimeric Tag Binding to *E. coli* Bacteria

*E. coli P4-NR (smooth)* bacteria were grown to O.D. = 1 and incubated with 0.22 or 0.022 mg/mL C3b-10-mer IK-Fluorescein complex for 30 min at 37 °C, 200 rpm. Three washes with PBS were performed. Each sample was placed on a thin layer of 1% agarose which was prepared on a microscope slide. Syto9 was added to the control sample. Images were obtained using an EVOS M5000 imaging system (Thermo Fisher Scientific).

### ELISA Assay

Biotin molecule was coupled to 10-mer IK and conjugated to human C3b protein (complement technology) using glutaraldehyde, as already described. Human C5 protein (complement technology) was dialyzed overnight against sodium bicarbonate buffer pH 9 and fixed on an ELISA plate in the concentration of 1 μM. Following that, it was incubated with 10-mer IK-biotin-C3b in serial dilution (highest concentration was 2.81 μM). The level of binding was assessed using α-streptavidin-HRP antibody in a dilution of 1:1000. TNB reagent was used with addition of 0.5 M H_2_SO_4_, and the absorbance at 420 nm was measured by a plate reader (Tecan).

### Serum Sensitivity Test

Both bacteria strains, *E. coli* strains *P4-NR smooth (E. coli S)* and P4-NRΔ*galU (E. coli R*), were grown to O.D. = 1 (10^8^ CFU/mL) in LB media and washed three times with PBS. The smooth bacteria strain was incubated together with 0.22 or 0.022 mg/mL 10-mer IK-C3b chimeric tag for 30 min at 37 °C, 200 rpm in PBS. After three washes with PBS, all samples were diluted to a concentration of 10^6^ CFU/mL. In a 24 well plate, 450 μL of bacteria in PBS were mixed together with 50 μL human serum. In every experiment, each sample had three wells which were dedicated for two types of serum, normal human serum (complement technology) and heat inactivated human serum for 30 min at 56 °C. The plate was incubated for 1 h at 37 °C. At the end of the incubation, microdilutions were made for each sample in 96 well plates; 10 μL drops were plated on LB agar plates and incubated overnight at 37 °C. In the same way, microdilutions and plating were done also at t = 0, before incubation.

## Supporting Information Available

Additional experimental data and mass spectrometry details; LC-MS/MS, MIC test, microscope images, and serum sensitivity tests (PDF)
