# Supplementary material for: Recruiting the Immune System against Pathogenic Bacteria Using High-Affinity Chimeric Tags
Source: Bioconjug Chem. 2024 Oct 14;35(11):1716–22. doi: 10.1021/acs.bioconjchem.4c00291 (PMC11583208; doi:10.1021/acs.bioconjchem.4c00291)
Supplement: Supplementary file 1 — bc4c00291_si_001.pdf [file bc4c00291_si_001.pdf]

Recruiting the immune system against pathogenic bacteria using high-affinity chimeric tags

Yael Belo, Einav Malach, Zvi Hayouka*

Institute of Biochemistry, Food Science and Nutrition, The Robert H. Smith Faculty of Agricultural, Food & Environment, The Hebrew University of Jerusalem, Rehovot, 76100, Israel.

zvi.hayouka@mail.huji.ac.il

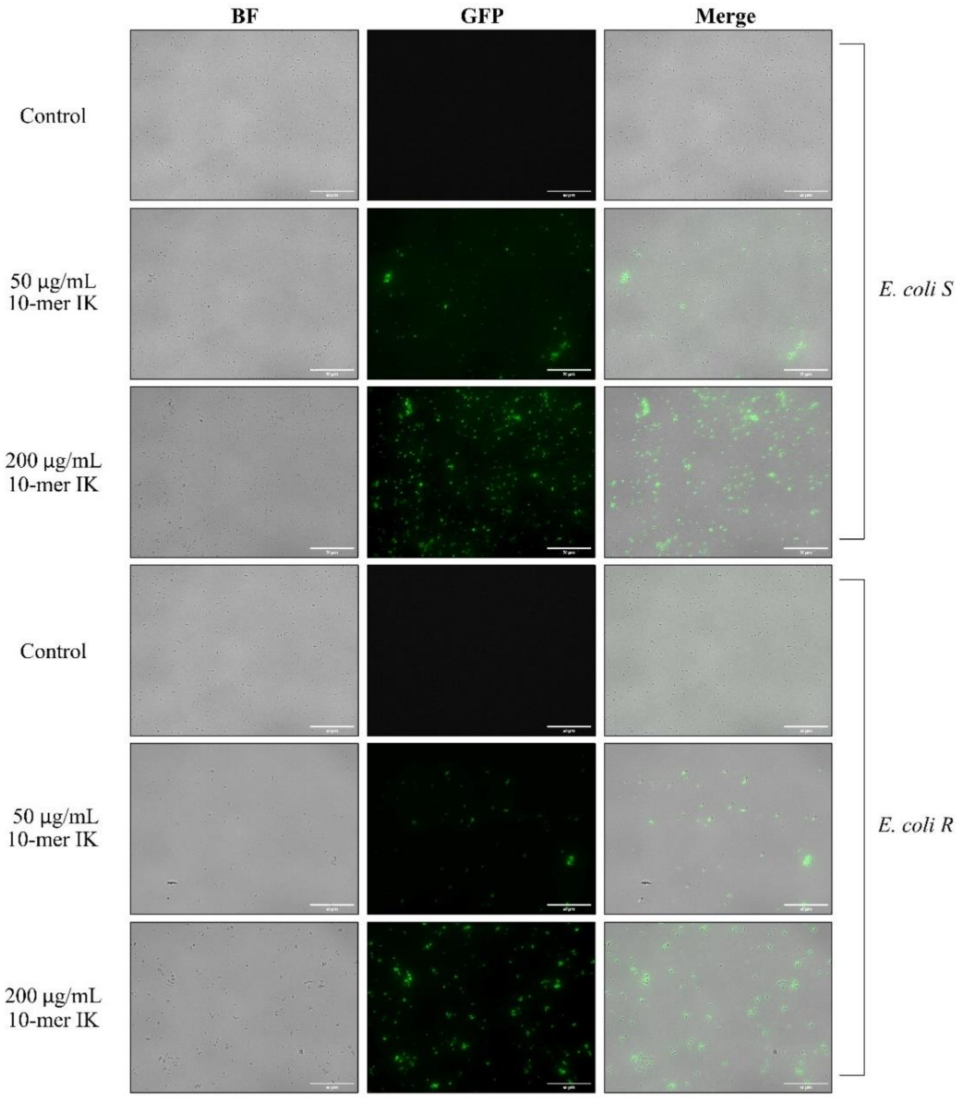

Figure S1: 10-mer IK binds *E. coli* bacteria. EVOS microscope images of bound 10-mer IK *E. coli* S and *E. coli* R bacteria. Images shown were taken at a magnification of 40×. Bacterial cells were grown to O.D. = 0.1, and incubated with fluorescently labeled peptide binder for 30 min at 37ºC in PBS.

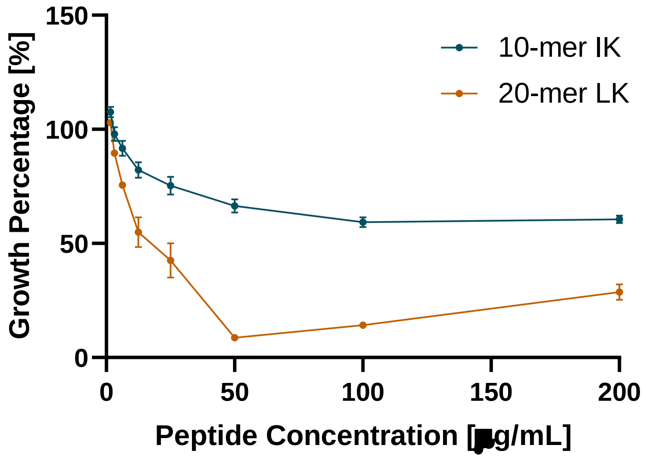

Figure S2: 10-mer IK RPM showed low antimicrobial activity towards *E. coli*. Growth-inhibitory activities of 10-mer IK and 20-mer LK random peptide mixtures, toward P4-NR *E. coli* bacteria, after the incubation of 24hr at 37°C. The experiments were repeated three times (biological repeats) in triplicates (average ± SEM).

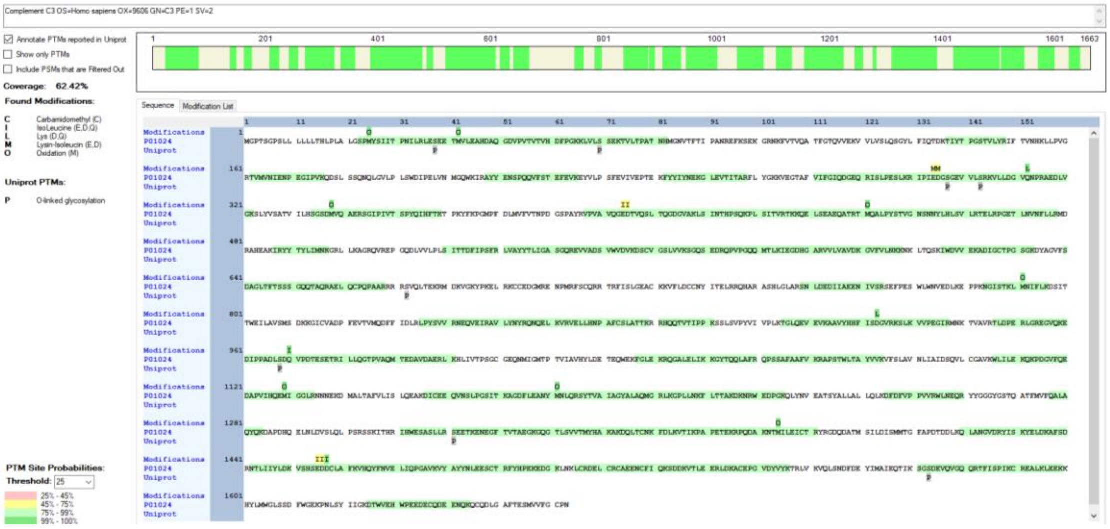

Figure S3: The C3b chimeric tag consists of several conjugated bacterial binder peptides. LC-MS/MS Analysis of C3b chimeric tag with conjugated bacterial binder peptides. The coverage map was generated using LC-MS/MS analysis following trypsin digestion. Peptides were identified using the Discoverer software against the human C3b sequence from the UniProt database. This map reveals the quantity and positions of the bacterial peptide units conjugated to the C3b protein. Each modification (I/L/M) indicates a 10-mer IK bacterial binder peptide conjugated to the C3b protein.

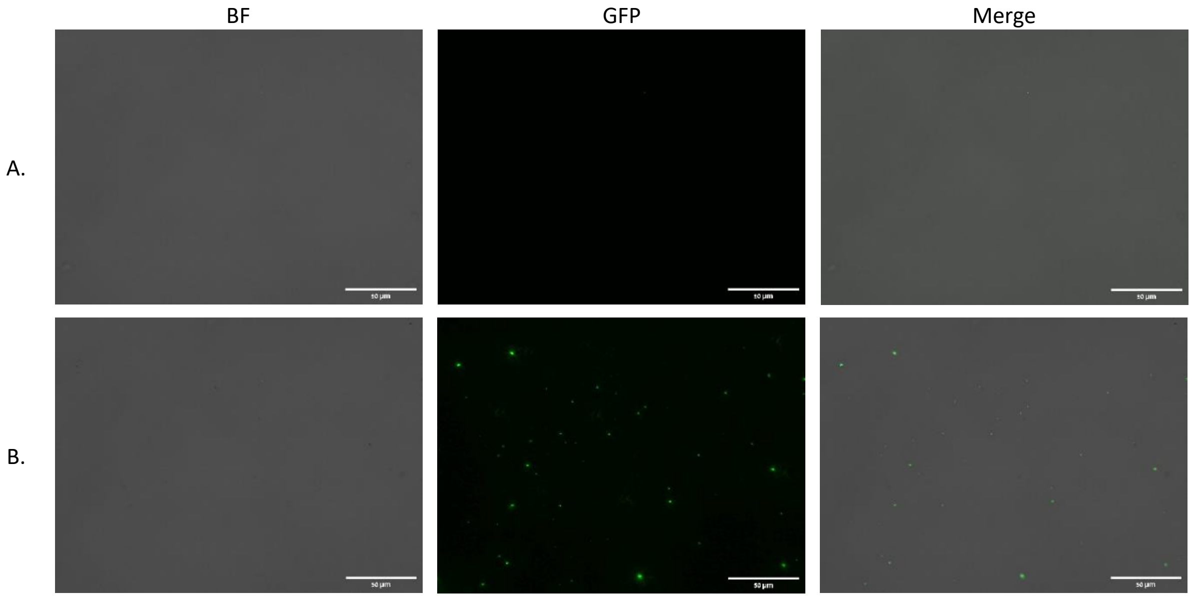

Figure S4: Fluorescent chimeric C3b tag does not form aggregates. EVOS microscope images of the chimeric C3b tag in PBS. A. PBS control sample. B. 0.22 mg/mL fluorescent chimeric tag in PBS. Images were taken at 40× magnification.

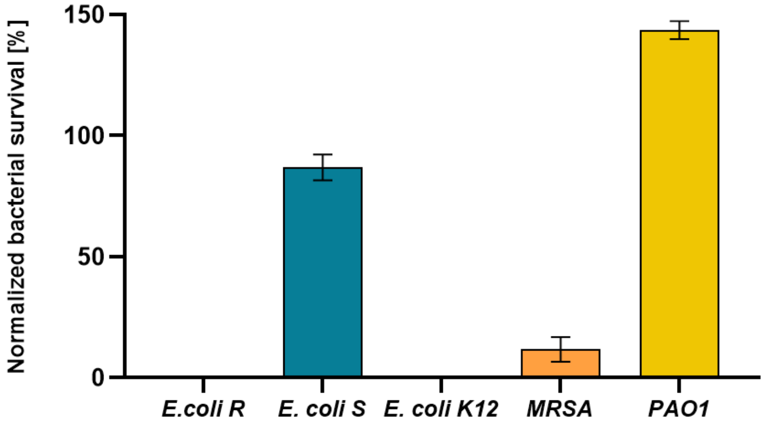

Figure S5: Screening of complement resistant pathogen bacteria. *E. coli P4-NR S, E. coli P4-NR R, E.coli K12, MRSA* and *PAO1* bacteria strains were incubated separately with normal bovine serum and inactivated serum for 1 hr at 37°C. Samples were micro diluted and plated on LB agar plates. The results show the percentage of bacteria that survived after 1 hour of incubation in normal serum, compared to the bacterial survival in inactivated serum. The average initial bacterial load was $5.44\times10^6\pm1.17\times10^6$ CFU/mL. Mean ± SEM (n=3).

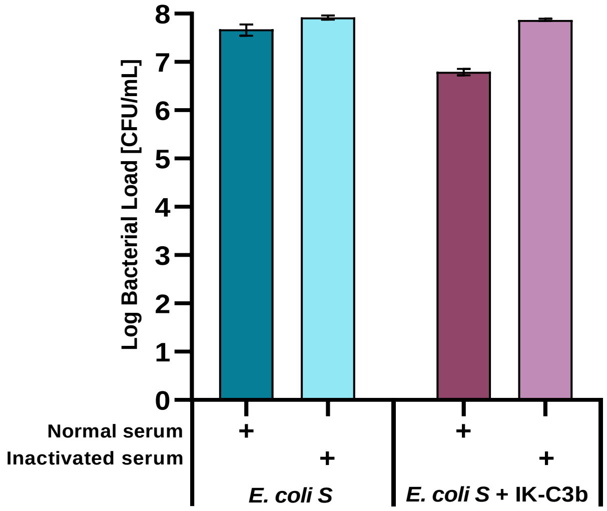

Figure S6: 10-mer IK-C3b complex induces complement sensitivity of *E. coli S* bacteria. *E. coli R, E. coli S* and *E. coli S* treated with the chimeric C3b tag, were separately incubated with normal human serum (dark color) or inactivated serum (light color) for 1 hr at 37°C. Samples were micro diluted and plated on LB agar plates. The results are displayed as the bacterial load after 1 hour of incubation. The average initial bacterial load was $2.93\times10^{6}\pm2.62\times10^{5}$ CFU/mL. This graph is a representative result chosen from a total of three biological repetitions. Mean ± SEM (n=3).
